# Novel neutralizing chicken IgY antibodies targeting 17 potent conserved peptides identified by SARS-CoV-2 proteome microarray, and future prospects

**DOI:** 10.3389/fimmu.2022.1074077

**Published:** 2022-12-22

**Authors:** Jin Li, Te Liang, Ailian Hei, Xiangbin Wang, Huijun Li, Xiaobo Yu, Rui Zhao, Peng Gao, Cong Fang, Ji Zhou, Maogang Li, Ellen He, Sven Skog

**Affiliations:** ^1^ Department of Medicine, Shenzhen Ellen-Sven Precision Medicine Institute, Shenzhen, China; ^2^ Beijing Key Laboratory for Forest Pest Control, Beijing Forestry University, Beijing, China; ^3^ SciProtech Co., Ltd, Beijing Changping Science Park, Beijing, China; ^4^ State Key Laboratory of Proteomics, Beijing Proteome Research Centre, National Centre for Protein Sciences-Beijing (PHOENIX Centre), Beijing Institute of LifeOmics, Beijing, China

**Keywords:** SARS-CoV-2, chicken egg yolk immunoglobulin (IgY), neutralizing IgY polyclonal antibodies (N-IgY-pAb), SARS-CoV-2 extracellular domain of the S-protein (S-ECD), residue mutations (RMs), neutralizing antibodies (NAbs)

## Abstract

**Introduction:**

An approach toward novel neutralizing IgY polyclonal antibodies (N-IgY-pAb) against SARS-CoV-2 S-ECD was developed.

**Material and methods:**

The novel N-IgY-pAb and its intranasal spray response against the wild type (“‘WH-Human 1”) SARS-CoV-2 virus, variants of Delta or Omicron were up to 98%. Unique virus peptides binding to N-IgY-pAb were screened by a SARS-CoV-2 proteome microarray.

**Results:**

Seventeen mutation-free peptides with a Z-score > 3.0 were identified as potent targets from a total of 966 peptides. The new findings show that one is in the RBM domain (^461^LKPFERDISTEIYQA^475^ ), two are in the NTD domain (^21^RTQLPPAYTNSFTRG^35^, ^291^CALDPLSETKCTLKS^305^) four are in the C1/2-terminal (^561^PFQQFGRDIADTTDA^575^,^571^DTTDAVRDPQTLEIL^585^,^581^TLEILDITPCSFGGV^595^, ^661^ECDIPIGAGICASYQ^675^ ), three are in the S1/S2 border (^741^YICGDSTECSNLLLQ^755^, ^811^KPSKRSFIEDLLFNK^825^, ^821^LLFNKVTLADAGFIK^835^) one target is in HR2 (^1161^SPDVDLGDISGINAS^1175^) and one is in HR2-TM (^1201^QELGKYEQYIKWPWY^1215^). Moreover, five potential peptides were in the NSP domain: nsp3-55 (^1361^SNEKQEILGTVSWNL^1375^), nsp14-50 (^614^HHANEYRLYLDAYNM^642^, ORF10-3 (^21^MNSRNYIAQVDVVNFNLT^38^, ORF7a-1(^1^MKIILFLALITLATC^15^) and ORF7a-12 (^1116^TLCFTLKRKTE^121^).

**Discussion and conclusion:**

We concluded that the N-IgY-pAb could effectively neutralize the SARS-CoV-2. The new findings of seventeen potent conserved peptides are extremely important for developing new vaccines and “cocktails” of neutralizing Abs for efficient treatments for patients infected with SARS-CoV-2.

## Introduction

Severe Acute Respiratory Syndrome Corona Virus 2 (SARS-CoV-2) causes significant morbidity and has involved a large number of medical consultations, hospitalizations, high medical and social costs. Now it is still spread fast all over the world and created a dramatic healthcare challenge for humankind (1–5). The earliest available SARS-CoV-2 viral genomes were identified from patients in December 2019, Wuhan, China, and as named “‘WH-Human 1” (2). The ongoing COVID-19 pandemic situation was caused by the SARS-CoV-2 and variants of concern such as B.1.617.2 (Delta) in India in late 2020. The B.1.1.529 (Omicron) is posing multiple challenges to humanity since 26 November 2021 until now (3). The Omicron (BA.1) variant with 50 mutations and approximately 32 of these pertain to the spike protein which most vaccines target to neutralize the virus. Many of the mutations are novel and are not found in previous variants of the virus (4, 5).

As is known, immunity can be acquired artificially by active vaccination and/or passive immunization (6–9). Because of the absence of specific antiviral drugs, it is critical that coronavirus specific vaccines must be developed. So far, there are a total of 320 vaccine candidates for SARS-CoV-2 at various stages of development, of which 194 are in preclinical stages, 126 in clinical development, and 9 have been approved *via* Emergency Use Authorization by different countries (6, 8).

SARS-CoV-2 consists of a 29.9 kb positive-sense single-stranded RNA genome. It encodes four structural proteins: nucleocapsid (N), membrane (M), envelope (E) and surface-anchored spike glycoprotein (1). The 29.9 kb sequence of full SARS-CoV-2 proteome has been deposited in the Global Initiative on Sharing All Influenza Data (GISAID). Antibodies against SARS-CoV-2 are divided into two main categories, neutralizing antibodies (NAbs) and non-neutralizing virus-binding antibodies (BAbs) (10). Since the SARS-CoV-2 virus invades its host *via* the interaction of its S-protein with the ACE-2 protein on the surface of host cells, the SARS-CoV-2 NAbs are raised against the S-protein. The crucial receptor-binding domain (RBD) localized to the S1 portion for the major target for human NAbs has been reviewed (11). On the other hand, the BAbs in most cases target the S-, N-, E- and Mproteins of the SARS-CoV-2 virus. Furthermore, the BAbs targets the N-protein are often used in the commercial detection tests to identify SARS-CoV-2 infected individuals (10).

A serious challenge currently facing the multiple SARS-CoV-2 variants is the frequent mutations of the virus, which can enhance its adaptability (1, 11–13). Those new mutant strains can easily cause repeated epidemics, weaken the protective effects of vaccines, or worsen the global spread of the epidemic (11, 13). Therefore, it is important to identify conserved peptides from the SARS-CoV-2 proteome for detection or treatment purposes, which enables avoidance of mutation sites, especially for extremely frequent mutation peptides. Data from GISAID show that 27 proteins of the SARS-CoV-2 virus are mutating at different rates, of which most exhibit little to no mutational variability. Specifically, the spike (S) and nucleocapsid (N) proteins exhibit the highest mutational variability, such as D614G (S), A222V and L18F (S), P323L (NSP12), R203K/G204R (N) and A220V (N) (1). From the Wilipedia website (https://en.wilipedia.org/wiki/sars-Covi-2_Delta_variant#Mutaions) and William’s report (13), the new Delta CoV-2 type showed mutations at D614G, T478K, L452R, P681R and E484Q (S).

**Figure 1:**
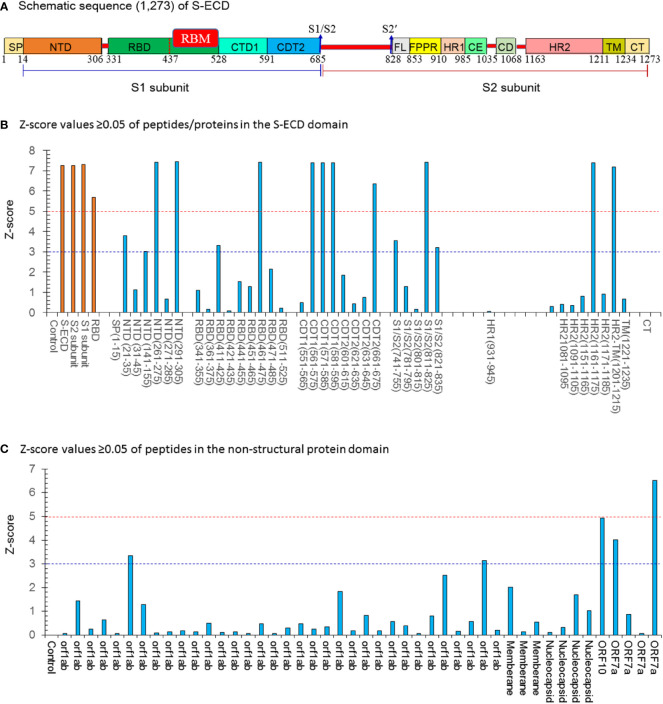
The Z-score values ≥0.05 of peptides/proteins in the S-ECD domain by the SARS-Cov-2 proteome microarray. **(A)** Schematic sequence (1,273) of a full-length SARS-CoV-2 spike. Two major domains within the spike protein are denoted as S1 head (S1) and S2 stalk regions (S2). The different domains are shown in different colors: SP (single sequence), NTD (N-terminal domain); RBD (receptor-binding domain) containing the RBM (receptor binding-motif), CTD1 (C-terminal domain 1), CTD2 (C-terminal domain 2), S1/S2 border (S1/S2 cleavage site), S2′ (S2′ cleavage site), FL (fusion loop), FPPR (fusion peptide proximal region), HR1(heptad repeat 1), CH (central helix region), CD (connector domain), HR2 (heptad repeat 2), TM (transmembrane domain), and CT (cytoplasmic tail). **(B)** Z-score values ≥0.05 of peptides/proteins in the S-ECD domain;15 potent peptides had a Z score ≥ 3.0. **(C)** Z-score values ≥0.05 of peptides/proteins in the non-structural protein domain. Five potent peptides had a Z-score ≥ 3.0. Con.: Control (PBS buffer).

Previous studies show that SARS-CoV-2 S protein is capable of triggering protease-independent and receptor-dependent syncytium formation. This enhances virus spreading through cell-cell fusion and rapid neutralizion of the virus (12). The extracellular domain of the S-protein (S-ECD) has denoted as S1 head (S1) and S2 stalk regions (S2) (see **Figure 1A**). NAbs to SARS-CoV-2 all target the receptor-binding domain (RBD), with blocking the ability of the RBD to bind human angiotensin-converting enzyme 2 (hACE2), while others bind core regions of the RBD to modulate spike stability or ability to fuse to host cell membranes. This is the main target for designing neutralizing antibodies (NAbs) and vaccines which play vital roles in studying the viral entry, determination of virulence, understanding the range of hosts, a pseudo-type system with S protein of SARS-CoV-2 (10–12, 14). With respect to the passive immunization strategy, specific immunoglobulins (IgY) extracted from egg-yolk of immunized hens have been employed to deal with virus infections (9).

Since the phylogenetic distance between mammals and birds causes a high immune response by birds to mammalian antigens, the immunoglobulin Y antibodies (IgYs) from the yolk of chicken eggs offer a series of advantages as compared to mammalian immunoglobulin G antibodies (IgGs) (14). In particular, IgYs have the potential to induce neutralizing antibodies against respiratory infections, can inhibit the cell-to-cell spread of virus particles, thus suppressing viral colonization, and possesses remarkable pathogen-neutralizing activity in the respiratory tract and lungs (15). The production of NIgY-pAb against selected antigens means high productivity, better animal welfare, higher immunogenicity, lower cross-reactivity, lower manufacturing costs, and easier storage (14). So far, an IgY pAbs has been developed using recombinant MERS-CoV S-subunit protein showing efficient neutralization both *in vitro* and *in vivo* with a MERS-CoV infection animal model (16). An IgY pAbs against the conservative nucleocapsid protein (NP) of SARS-CoV-2 showed strong NP binding ability (17). IgY pAbs against the SARS-CoV-2 S-protein showed two high signals in the NTD domain and

S1/S2 border using analysis of proteome microarray (18). However, it is still unknown whether IgY Abs are consistently able to present top responses against peptides containing the specific epitope of the SARs-CoV-2.

In this study, we developed a novel neutralizing IgYAb (N-IgY-pAb) against the SARS-CoV-2 extracellular domain of the S-protein (S-ECD). The full-length S-ECD is composed of 1,273 amino acids and assembled into a homo-trimeric complex (spike trimer protein) to increase its capacity to elicit neutralizing antibodies (19). Characterization of the N-IgY-pAb targeting 966 peptides was performed on a SARS-CoV-2 proteome microarray system (20). Seventeen of these peptides did not show any mutations, with a Z-score > 3.0, and thus were potent peptides as targets for immunetreatment. The new findings of seventeen potent conserved peptides are extremely important for developing new vaccines and “cocktails” of neutralizing Abs for the efficient treatment of SARSCoV-2.

## Methods

### Immunization of chickens

A purified recombinant SARS-CoV-2 S-ECD protein (S-ECD) (Catalog number: Z03481) was obtained from GenScript USA, Inc. (Piscataway, NJ, USA) with a His Tag & Flag Tag at the C-terminus expressed in Sf9 insect cells (Val16-Pro1213; GenBank No. QHD43416.1). In brief, 25-week-old Leghorn hens were immunized intramuscularly with 250 μg purified recombinant S-ECD protein mixed with an equal volume of complete Freund’s adjuvant (Sigma-Aldrich, USA) at five different sites of thigh muscles in the initial injection. Then, immunization with 125μg S-ECD protein mixed with an equal volume of incomplete Freund’s adjuvant was conducted two weeks later. Subsequently, two injections of 25 μg purified recombinant SARS-CoV-2 S-ECD were applied to enhance immunization every two weeks for two months. Eggs were collected one week after the final injection and labelled with the date and hen ID, and then stored at 4°C until antibody extraction. Meanwhile, eggs from non-immunized hens were collected as negative controls. All methods in this study were performed in accordance with the relevant guidelines and regulations.

### Extraction and purification of N-IgY-pAb

N-IgY-pAbs were extracted and purified in our lab (14). Briefly, the egg yolk was separated from the white and washed with distilled water to remove albumen. The washed egg yolk was then diluted with distilled water (acidified with 0.1N HCl), and held for at least two hours, then centrifuged at 12,000 r/min at 4°C for 25 min. The supernatant was carefully transferred, and 19% (W/V) ammonium sulphate was slowly added into the supernatant solution. After thorough mixing at room temperature for two hours, the mixture was centrifuged at 12,000 r/min for 25 min at 4°C. After removal of the supernatant, the pellet was dissolved into phosphate buffered saline (PBS; pH 7.4) followed by centrifugation at 12,000 r/min for five minutes at 4°C, then the supernatant was collected. The N-IgY-pAbs in the supernatant were purified by an S-ECD affinity column and then determined by a reduction SDS polyacrylamide gel electrophoresis (SDS-PAGE).

### N-IgY-pAb targeting S-ECD and RBD by ELISA assay

Three different batches of N-IgY-pAb targeting S-ECD and RBD were analyzed in our laboratory by ELISA. Briefly, the IgY polyclonal antibody against SARS-CoV-2 S-ECD and RBD was evaluated using an enzyme-linked immunosorbent assay (ELISA). The 96-well plates (Catalog number: 456529, Thermo Fisher Scientific, USA) were coated with 100 μL S-ECD- or RBD protein per well at 0.5μg/mL in carbonate buffer (pH 9.6) and incubated overnight at 4°C. The wells were rinsed once, and a blocking buffer was added (5% nonfat milk in PBS) for one hour at 37°C. The wells were rinsed, and then the purified N-IgY-pAbs with different concentrations (3.12–200 ng/mL) were added into the wells and incubated for one hour at 37°C. After three rounds of washing, the plate was incubated with 1:10,000 HRP-conjugated goat IgG for one hour at 37°C followed by three rounds of washing. Tetramethylbenzidine (TMB; 100 μL per well) was added and incubated at 37°C for 10 minutes. 100 μL 1M H_2_SO_4_ was added to each well to terminate the reaction. The plates were then scanned in a spectrophotometer and the absorbance was read at 450 nm.

### The preparation of N-IgY-pAb spray and stability evaluation

The IgY-Ab from the native yolk eggs of laying hens is stable (9, 15), safe (9) and does not trigger potentially dangerous immune responses (16). The IgYs have previously been used against bacterial and viral infections in humans and animals. In this study, a nasal spray containing N-IgY-pAb was prepared. Briefly, in a sterile environment, the N-IgY pAb antibody extract was sterilized through filtering devices, then was dissolved in sterile 0.9% sodium chloride solution (21). The final concentration of N-IgY pAb antibody was adjusted to 6.0 ug/mL. Then, the solution was packed into a nasal spray device as 30mL/bottle for use. The stability of the N-IgY-pAb in the spray was tested using the accelerated thermal stability method at 37 °C for 0–7 days, or storage at ≈4°C for 6–12 months (9, 15, 21). The stability was evaluated by ELISA.

### N-IgY-pAb response against authentic SARS-CoV-2

In order to determine the neutralization effect of the N-IgY-pAb, growing Vero cells *in vitro* were used. The N-IgY-pAb against SARS-CoV-2 was tested on wild type virus (“‘WH-Human 1”), and on variants (B.1.617.2, Delta; B.1.1.529, Omicron) in three Biosafety Level 3 Laboratories (BSL-3) (Center of Disease Control, Shenzhen, China; the Key Laboratory of Virology, Wuhan University; the Wuhan Institute of Biological Products Co. LTD, China), respectively.

#### Determining the number of copies of the virus RNA by real time RT-PCR (reverse transcription-polymerase chain reaction)

The cells were treated with a mixture of N-IgY-pAb antibody and authentic SARS-CoV-2 virus. Cells only treated with the SARS-CoV-2 virus were used as controls. Briefly: **1)** Vero cells (10^5^/mL) in DMEM medium (2% FBS in DMEM with penicillin/streptomycin) were seeded into 96-well plates (100 μL per well) and incubated overnight at 37°C in a CO_2_ incubator. The virus infection was performed when the cell density reached 80–90%. **2)** A mixture of serially diluted purified N-IgY-pAb or “intranasal spray” with SARS-CoV-2 virus (Median Tissue Culture Infectious Dose (TCID_50_)) were added to the cells and incubated at 37°C for one hour (100 cells in 100 μL total). Cells growing in DMEM or PBS without the virus were used as controls. **3)** The medium was removed and 100 μL of the DMEM medium without N-IgY-pAb and SARS-CoV-2 virus was added and incubated at 37 °C for further 48 hours. **4)** The DMEM medium was removed, and the total RNA of the cells was extracted using Roche’s High Pure Viral RNA Purification kit, USA (# 11858882001) according to the method described by the manufacturer. **5)** The copy number of SARS-CoV-2 RNA in the total RNA was detected by using Shanghai Berger’s Coronavirus Nucleic Acid Detection kit (Catolog number: NMPA20203400065). The nucleic acid concentration in the virus sample was determined by a fluorescence quantitative PCR method based on a formula including the Ct value (number of PCR cycles giving detectable values) of the open reading frame 1 ab (ORF1ab) and the nucleocapsid protein (N-gene) (fluorescence quantitative PCR, Shanghai Berger Medical Technology Co., Ltd. China). The primers and probes for the SARS-CoV-2 virus used to target both the ORF1ab and N-gene (ROCHE, High Pure Viral RNA Kit, #11858882001). The data were plotted after background subtraction and normalized to the controls. The virus-neutralizing effect was accepted when the neutralizing rate was ≥ 95% (22).

#### Live virus focus-reduction neutralization test

The FRNT assay was carried out in the BSL-3 of the Shenzhen Third People’s Hospital, China. The “intranasal spray” was serially diluted and incubated with SARS-CoV-2 variants (Delta variants: B.1.617.2 and Omicron variants:EPI_ISL_11799984), respectively for 1 hour at 37 °C. Each concentration was replicated in three wells. The mixture of virus and N-IgY-pAb were added to the seeded Vero E6 cells and incubated for 1 hour at 37 °C. The protocol of FRNT was followed as previously described by Zhou, et al., 2022 (23). Finally, the number of spots was calculated by reading the plates with an ELISpot reader with the chromogen deposit readout. The neutralizing ratio = (1-the spots in well with N-IgY-Ab/the spots in well without N-IgY-Ab) x100%. The virus-neutralizing effect was accepted when the neutralizing rate was ≥ 95% (22).

### SARS-CoV-2 proteome microarray system assay

The mapping of the N-IgY-pAb binding peptides or proteins of SARS-CoV-2 protein was performed using a SARS-CoV-2 proteome microarray system as described previously (20). Four arrays were prepared, two serums with N-IgY-pAb and two serums from pre-immunized hen (as control). Briefly: The proteome microarrays were prepared with tray of 2,018 spots. All biotin-labelled peptides were obtained from China Peptides, Guoping Pharmaceutical Company (Beijing, China).

All SARS-CoV-2 N-, and S-proteins were obtained from Sino Biological, Inc. The peptides and proteins were printed onto a 3D modified slide surface in duplicate using an Arrayjet microarrayer (20). The microarrays were stored at −20°C until ready to use.

For evaluating the Immune response of the N-IgY-pAb, four microarrays were incubated in parallel and blocked with PBS containing 5% (w/v) milk with 0.05% (v/v) Tween-20 (PBST) for 10 min at room temperature. After washing, two microarrays were incubated with the purified N-IgY-pAb (microarray 1^#^ with 375 ng/mL and microarray 2^#^ with 186 ng/mL, respectively). In the meantime, the pre-immunization hen’s serum was added to microarrays 3^#^ (serum dilution 2000x) and microarray 4^#^ (serum dilution 4000x). The four microarrays were running for 30 min at room temperature.

Subsequently, following three rounds of washing, the array was incubated with goat anti-chicken secondary antibody (Catalog number: ab150170, Abcam, Waltham, MA. USA) labeled with Alexa Fluor 555 for 20 min at room temperature. The arrays were washed, dissembled from the tray, and then dried with a vacuum pump.

The array was scanned through a GenePix 4300A microarray scanner (Molecular Devices, San Jose, CA, USA). The median intensity of the fluorescent signal of each spot was extracted using the GenePix Pro7 software (Molecular Devices, San Jose, CA, USA). The raw fluorescence signal intensity was the median signal intensity subtracted by the median background intensity of each spot, and then averaged across duplicate spots. The resulting signals were normalized with a Z-score, described by Wang et al. (20).

### Statistical analysis

The statistical analysis of the T-test (2-tailed) was performed using the statistical program SPSS 25.0 (IBM Corp., NY, USA) for descriptive data interpretation. P < 0.05 was considered to indicate a statistically significant value.

## Result

### The purity and immunoreactivity of N-IgY-pAb

**Figure 2:**
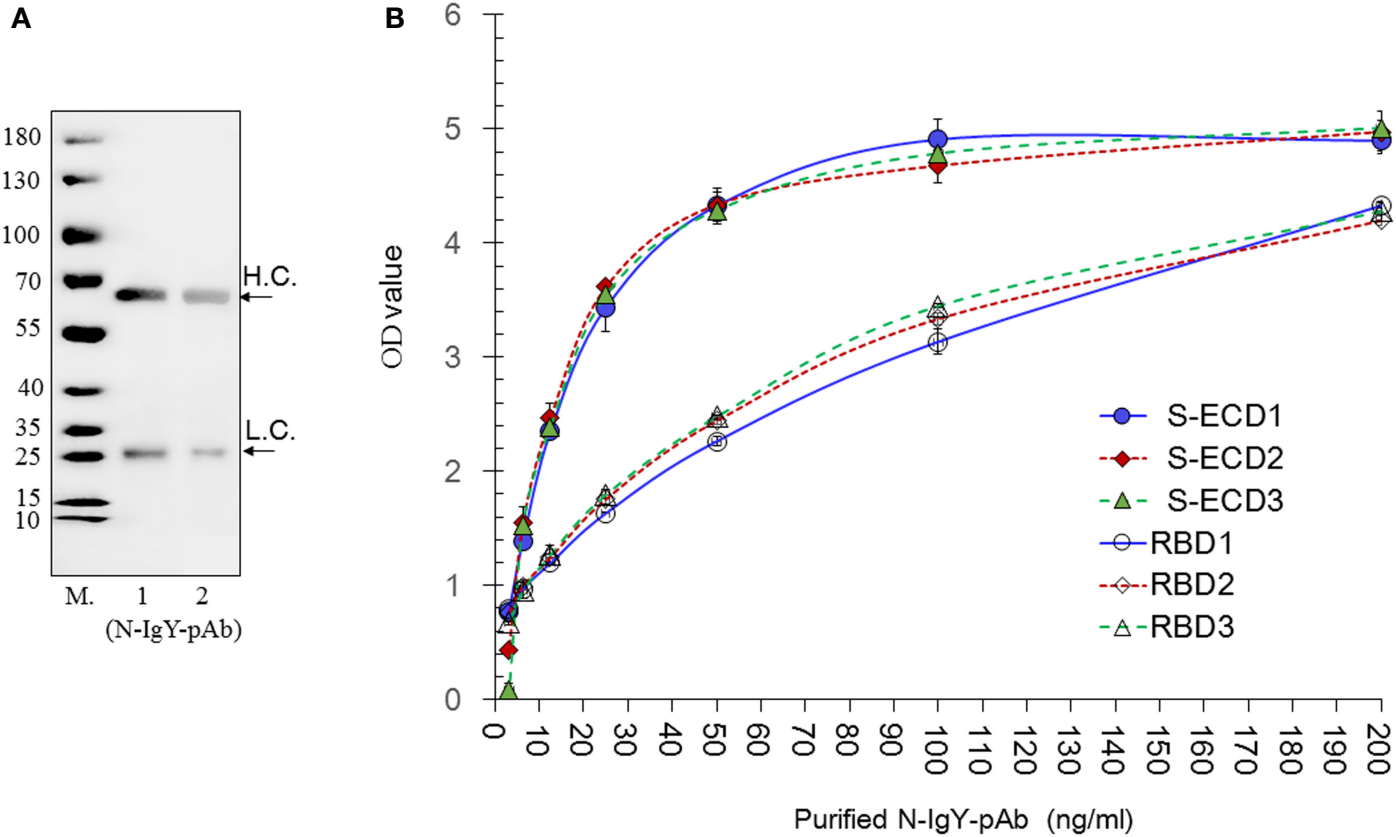
Evaluation of the purity, sensitivity and specificity of the N-IgY-pAb. **(A)** SDS PAGE of the N-IgY-pAb. The molecular weight (M) of the N-IgY-pAb were analyzed on a 12% SDS-PAGE. Lane M (Protein marker); lane 1: 2.5μg N-IgY-pAb; lane 2: 1.25μg N-IgY-pAb. **(B)** The immune activities of the N-IgY-pAb. S-ECD- or RBD protein (0.5μg/mL) using ELISA titer analysis. Thee three batches of purified N-IgY-pAb with a dilution of 200–3.12 ng/mL, respectively. H.L.= heavy chain; L.C. = light chain. H.L.= heavy chain; L.C. = light chain. The mean ± SD was from each sample in replicate two times.

The purity of the N-IgY-pAb was assessed by running a reducing SDS PAGE (**Figure 2A**) presenting two single bands corresponding to heavy chain (H.C.) and light chain (L.C.) according to the IgY’s molecular weight of about 180 kD (24).

The immune reaction against S-ECD and RBD from the purified N-IgY-pAb was determined by a dilution ELISA test (3.12-200 ng/ml). The results are shown in **Figure 2B**. The OD value below 40 ng/mL shows a linear increase (p<0.01), while no significant differences were observed when the concentrations of the N-IgY-pAb were above 40 ng/mL (p>0.05). No difference was found between the three batches of N-IgY-pAb tested (p>0.05: ≈0.25–0.96). Thus, an excessive antibody concentration of 100–200 ng/mL should be used when assessing the immune activities in the ELISA assays.

### Evaluation of the stability of the N-IgY-pAb in the “intranasal spray”

The stability of the N-IgY-pAb in the “intranasal spray” was tested by using an accelerated thermal stability method (37°C, 0–7 days, **Table 2A**) or by storage at 4°C for 6–12 months (**Table 2B**). Both tests confirmed that the N-IgY-pAb binding activity did not decline significantly over time, indicating that the N-IgY-pAb are stable for at least 12 months at 4°C.

### N-IgY-pAb response against authentic SARS-CoV-2

**Figure 3:**
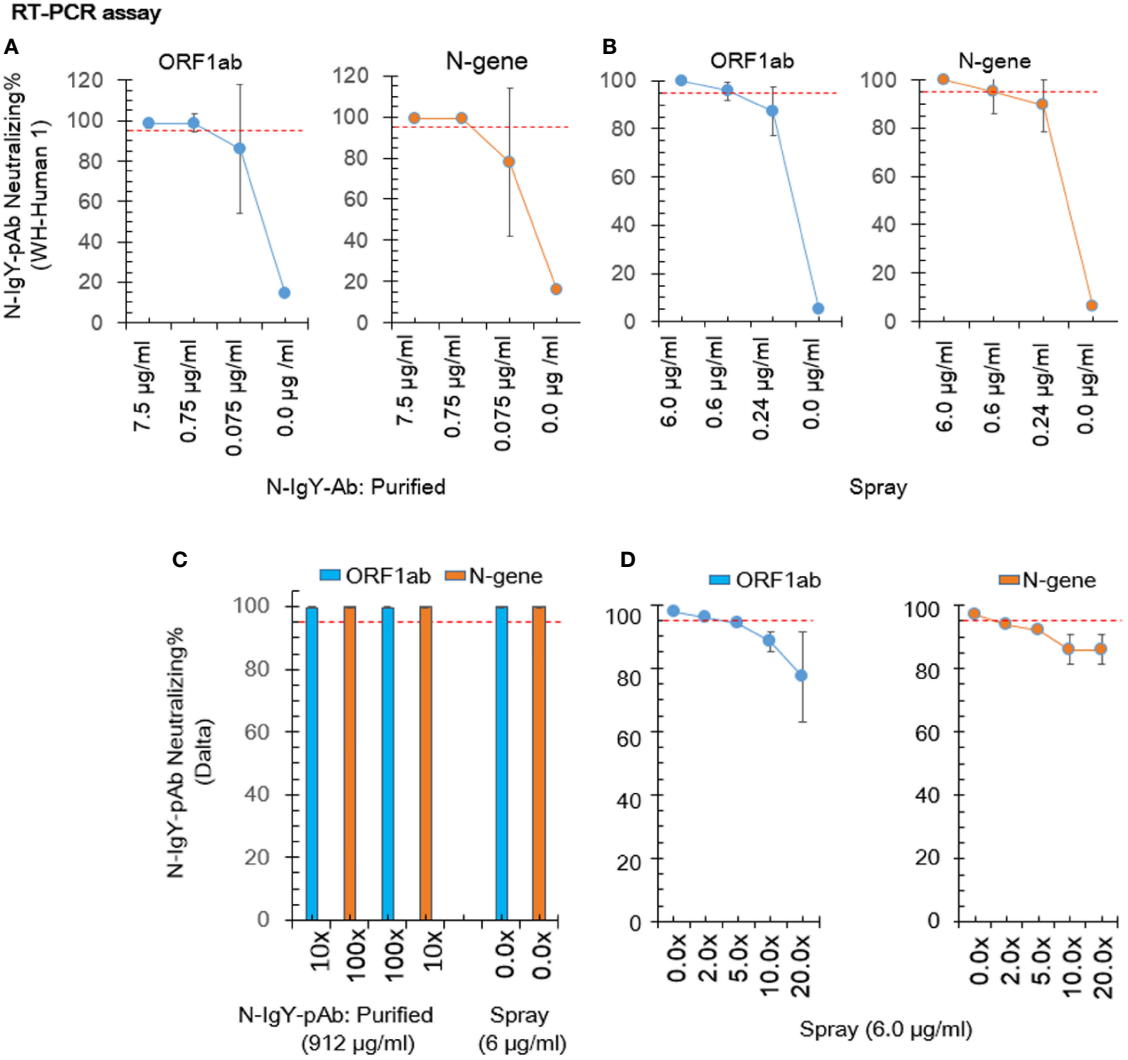
Neutralization test by RT-PCR. The nucleic acid concentration in the virus sample was calculated based on a formula including the Ct value (number of PCR cycles giving detectable values) of the open reading frame 1ab (ORF1ab) and the nucleocapsid protein (N-gene). The purified N-IgYpAb **(A)** or novel “spray” **(B)** against WH-Human 1 was carried out in the Center of Disease Control, Shenzhen, China. The “spray” against SARS-CoV-2 variants B.1.617.2 (Delta) and B.1.1.529 (Omicron) were carried out in the Key Laboratory of Virology, Wuhan University China **(C)** and the Wuhan Institute of Biological Products Co. LTD, China **(D)**, respectively. The mean ± SD was from each sample in replicate three times. The neutralization potential has to be achieved ≥ 95% (red dotted line).

**Figure 4:**
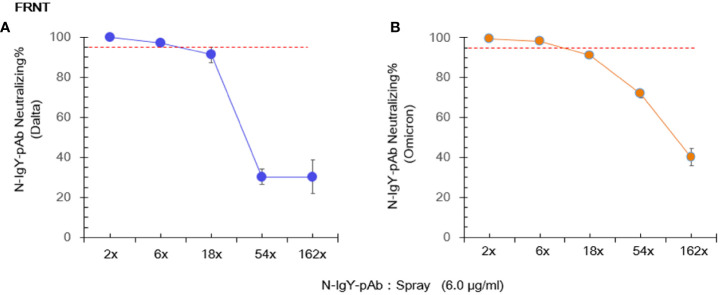
Live virus focus-reduction neutralization test (FRNT) involves immunostaining of virus infected cells with a chromogen deposit readout. The novel “spray” against the virus variants (Delta: B.1.617.2 **(A)** and Omicron: EPI_ISL_11799984 **(B)** was run in Shenzhen Third People’s Hospital, China. The mean ± SD was from each sample in replicate three times. The neutralization potential has to be achieved ≥ 95% (red dotted line).

We cooperated with three independent BSL-3 laboratories. Results of the RT-PCR assay and the FRNT assay are presented in **Figures 3A–D** and **Figures 4A, B**, respectively. The 0.75 μg/ml of purified novel N-IgY-pAb and its “intranasal spray” containing 6 µg/ml responded potent against the wild type SARS-Cov-2 virus (WH-Human 1), the variants of Delta and Omicron up to 98%. The results from the FRNT assay confirmed the RT-PCR assay. The novel N-IgY-pAb “intranasal spray” at 3 μg/mL (2x dilution, **Figures 4A, B**) responded against the wild type of the SARS-CoV-2 virus, the variants of Delta and Omicron, corresponding to the neutralizing rate of ≥ 95% (22). It implied that the “intranasal spray” containing 6μg/ml of N-IgY-pAb could be adequate to neutralize the virus effectively.

### The N-IgY-pAb binding sites using proteome microarray

According to a previous study (20), spots with a Z-score > 3.0 were evaluated as significantly strong signals. The spots with a Z score ≥ 5.0 were assessed as the highest response signals. The optimal concentration of the purified N-IgY-pAb for the proteome microarray was 375 ng/mL and the preimmunization hen’s serum was diluted at a ratio of 1:2000. The microarray was scanned through a microarray scanner and used the original raw fluorescence signal intensity of each spot. The resulting signals were normalized with the Z-score (mean value of duplicate spots).

**Figure 5:**
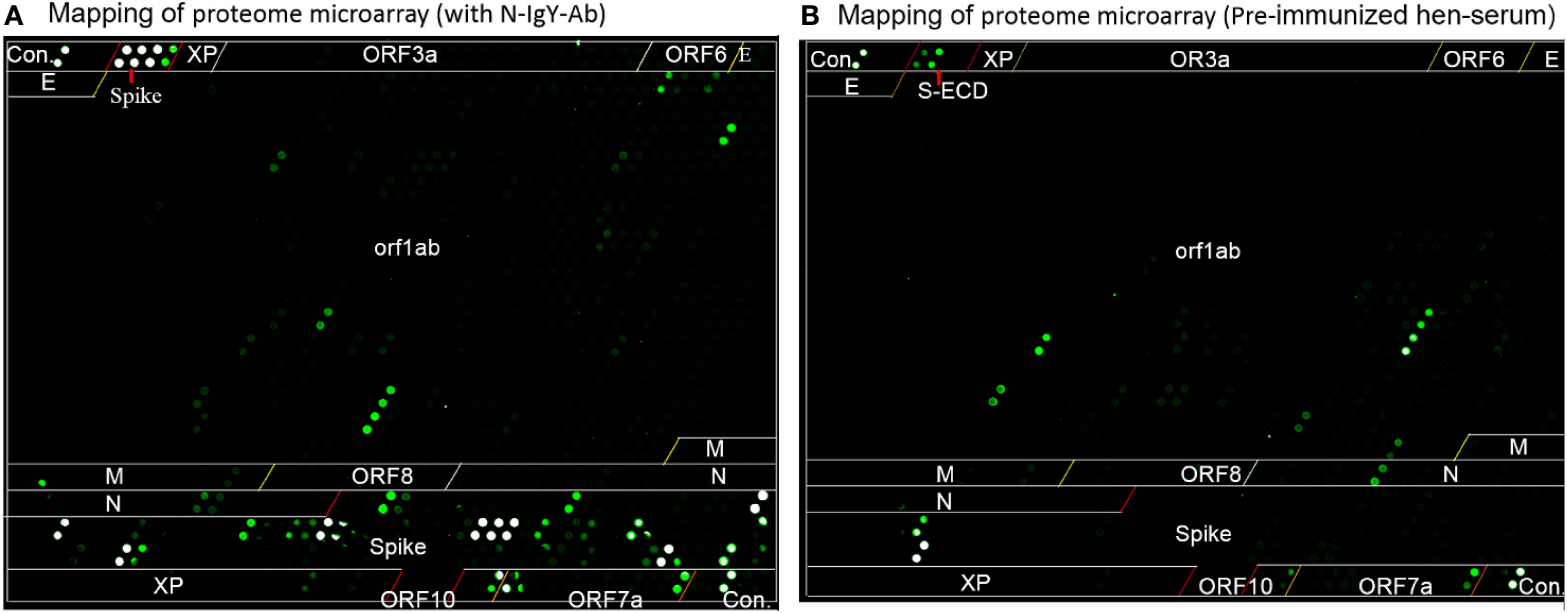
The mapping of proteome microarray. **(A)** the purified N-IgY-pAb binding peptides or proteins of SARS-CoV-2 protein, **(B)** Serum of the pre-immunized hen.

The microarray scanner of the array showed the original raw fluorescence signal intensity in **Figures 5** **A, B**. The four negative controls in a total of six controls showed negative signals when a positive signal with the mix of human IgG, IgM and IgA was presented. The human polio peptide was commonly used for a positive control for testing mAb (IgG). However, the N-IgY-pAb did not recognize the human polio peptide.

The N-IgY-pAb targeting 20 potent peptides were identified as showing significantly strong signals (Z-score> 3.0) and 11 peptides had a Z-score ≥ 5.0 (**Figures 1A–C**, **Table 1**). It should be noted that seventeen mutation-free peptides among the 20 peptides with a Z-score > 3.0 were identified as potent targets from a total of 966 peptides, shown in **Figure 2B** and **Table 1**, **Figures 1A–C** and **Table 1**, one is in the RBM domain ^(461^LKPFERDISTEIYQA^475^), two are in the NTD domain (^21^RTQLPPAYTNSFTRG^35^, ^291^CALDPLSETKCTLKS^305^), four are in the C1/2terminal (^561^PFQQFGRDIADTTDA^575^, ^571^DTTDAVRDPQTLEIL^585^, ^581^TLEILDITPCSFGGV^595^, ^661^ECDIPIGAGICASYQ^675^), three are in the S1/S2 border (^741^YICGDSTECSNLLLQ^755^, ^811^KPSKRSFIEDLLFNK^825^, ^821^LLFNKVTLADAGFIK^835)^, one target is in HR2 (^1161^SPDVDLGDISGINAS^1175^) and one is in HR2-TM (^1201^QELGKYEQYIKWPWY^1215^). Moreover, five potential peptides are in the NSP domain: nsp3-55 (^1361^SNEKQEILGTVSWNL^1375^), nsp14-50 (^6144^HHANEYRLYLDAYNM^6425^), ORF10-3 (^21^MNSRNYIAQVDVVNFNLT^38^), ORF7a-1

**Table 1:** Binding sizes of N-IgY-pAb to peptides or proteins of S-ECD in SARS-CoV- 2 virus.

Sample No	Z-score ≥ 3.0				
M. No.	N-IgY-pAb	Pre-immuno serum	residues	*NR	domain
S-ECD					
7	7.3	6.09			S1+S2
8	7.27	10.23			S2
9	7.25	0.54			S1
10	5.68	0.33			RBD
Peptide					
Non-structural proteins (NSP)					
190	3.35	<0.05	SNEKQEILGTVSWNL	15	orf1ab- 137
695	3.15	<0.05	HHANEYRLYLDAYNM	15	orf1ab-642
S-ECD					
840	3.78	<0.05	RTQLPPAYTNSFTRG	15	NTD (21-35)
852	3.01	0.15	LGVYYHKNNKSWMES	15	*NTD (141- 155)
864	7.41	<0.05	GAAAYYVGYLQPRTF	15	*NTD (261-275)
867	7.45	<0.05	CALDPLSETKCTLKS	15	NTD(291-305)
879	3.31	<0.05	APGQTGKIADYNYKL	15	*RBD (411-425)
884	7.42	<0.05	LKPFERDISTEIYQA	15	RBD(461-475)
894	7.38	0.192	PFQQFGRDIADTTDA	15	CTD1(561-575)
895	7.38	<0.05	DTTDAVRDPQTLEIL	15	CTD1(571-585)
896	7.38	<0.05	TLEILDITPCSFGGV	15	CTD1(581-595)
904	6.34	<0.05	ECDIPIGAGICASYQ	15	CTD2(661-675)
912	3.56	<0.05	YICGDSTECSNLLLQ	15	S1/S2(741-755)
919	7.43	14.16	KPSKRSFIEDLLFNK	15	S1/S2(811-825)
920	3.19	<0.05	LLFNKVTLADAGFIK	15	S1/S2 (821-835)
954	7.38	<0.05	SPDVDLGDISGINAS	15	HR2(1161-1175)
958	7.19	<0.05	QELGKYEQYIKWPWY	15	HR2-TM (1201-1215)
Non-structural proteins (NSP)					
991	4.93	1.46	MNSRNYIAQVDVVNFNLT	18	ORF10-3
992	4.01	< 0.05	MKIILFLALITLATC	15	ORF7a- 1
1003	6.52	6.46	TLCFTLKRKTE	11	ORF7a- 12
Controls					
1	positive	< 0.05	KEVPALTAVETGAT		polio peptide
2	negative	< 0.05	YPYDVPDYAG		HP
3	positive	7.132			Human IgG+IgM+IgA
4	negative	< 0.05			Streptavidin
5	negative	< 0.05			Buffer PBS
6	negative	< 0.05			N-protein

• Mutation: Grey box *NTD (141LGVYYHKNNKSWMES155, (G142D); *NTD.

(261GAAAYYVGYLQPRTF275); *RBD (411APGQTGKIADYNYKL425, (K417N). Comparative analysis of the immune response of the N-IgY-pAb and non- immunized hen serum targeting sites of the peptides in **Figures 5A, B** was summarized. *NR, number of residues; HP, hemagglutinin peptide.

**Table 2:** The stability of the N-IgY-pAbs in spray solution for 0-7 days at 37°C (A) and 0-12 months at 4°C (B).

A.							
N-IgY-pAbs,	RBD, 0.5μg/mL				S-ECD, 0.5μg/mL		
	0 day		7 day		0 day		7 day
Mean	5.24		5.21		5.23		5.21
STDEV	0.14		0.16		0.08		0.14
CV	2.61%		3.16%		1.46%		2.71%
T-test			0.480				0.607
B.							
N-IgY-pAb	RBD, 0.5μg/mL				S-ECD, 0.5μg/mL		
	0 m.	6 m.		12 m.	0 m.	6 m.	12 m.
Mean	5.27	5.64		5.40	5.43	5.59	5.58
STDEV	0.27	0.38		0.34	0.40	0.22	0.40
CV%	5.12%	6.74%		6.30%	7.37%	3.93%	7.17%
T-test		0.073		0.190		0.234	0.241
Control (PBS)	0.18	ND		0.16	0.17	ND	0.12

m, month The stability was tested in an ELISA coated by RBD or S-ECD. The mean and SD values were based on 20 repeated tests at the storage time from day 0 to day 7 at 37°C, or at the storage time from 0 to 12 months at 4°C. The values were expressed as OD.

(^1^MKIILFLALITLATC^15^) and ORF7a-12 (^111^TLCFTLKRKTE^121^). We concluded that the N-IgY-pAb could effectively neutralize the SARS-CoV-2. Only three peptides with mutation of amino acid residue were found (**Table 1**).

It should be noted that four significantly strong signals (S1+S2, S2 protein, S1/S2 (811–825), and ORF7a-12, Z-score > 3.0) were found in the serum of the pre-immunized hen (**Figure 5B**, **Table 1**). A likely explanation is that the pre-immunized hen’s serum carrying its own neutralizing antibodies recognizes some peptides that are similar to the SARS-CoV family. The IgY antibodies can neutralize the virus by several mechanisms – by blocking the attachment of the virus to the host tissue; preventing the membrane fusion or promoting the detachment of bound virus; interfering with the free virus; or causing aggregation of virus particles resulting in virus immobilization (15, 25, 26).

In addition, Lu et al. reported that they prepared IgY pAbs raised against S protein; primary purification of the IgY antibody was initially extracted by polyethylene glycol (PEG) precipitation, and then the IgY epitope mapping using the SARS-CoV-2 proteome microarray was performed (18). The results showed that the IgY pAbs targeted two epitopes (NTD domain: LDPLSET, and S1/S2 border: SIIAYTMSL) with high signals and three with relatively weak signals (AIHADQL and QIYKTPP (S1/S2 border) and DLGDISGIN (HR2) using the SARS-CoV-2 proteome microarray (18). It was surprising to us that no signal was found in the RBD domain in the study of Lu et al. According to our experience, the supernatant from PEG from the initial purification contained multi- and non-specific antibodies. It is necessary to further purify the IgY antibodies using the S-ECD affinity column (see Method). Generally speaking, only 2–3% of high-affinity purified antibodies identify special targets (peptides or epitopes) in the S-ECD protein. Therefore, the unexpected result may be due to a mixture of multi-antibodies.

## Discussion

Based on immunological, previous experimental, and clinical knowledge of the pathogenesis of SARSCoV-2 and its mutation, as well as the MERS respiratory syndrome, the passive immunization of neutralizing antibodies (NAbs) is a promising therapeutic and preventive treatment when combining with therapeutic vaccines against SARS-CoV-2 virus (10, 11). In this study, we addressed the use of passive chicken neutralizing IgY-polyclonal antibodies (N-IgY-pAb), since the novel N-IgY-pAb and its “intranasal spray” neutralized the wild type of SARS-CoV-2 (“‘WH-Human 1”) and variants of Delta and Omicron up to 98%. The N-IgY-pAb are very stable in storage at ≈4°C for at least one year. A new finding is that the N-IgY-pAb target 17 potential peptides, showing no residue mutations. This will be useful for the detection and treatment of the SARS-Cov-2 virus.

In order to identify specific binding sites, affinity-purified N-IgY-pAb targeting 966 peptides were tested in this study using a SARS-Cov-2 proteome microarray mapping (**Figure 1B, C** and **Table 1**). The new finding is that 20 peptide probes with significantly strong signals (Z-score > 3.0) were identified in a total of 966 peptides. The residues of ^461^LKPFERDISTEIYQA^475^ in the RBD domain presented the highest signal (Z-score = 7.42). A verified residue 438−498 within the RBD domain can directly engage the ACE2 receptor (27). It supports why the novel N-IgY-Ab is potentially effective in inactivating the SARS-CoV-2 virus (>98%) (**Table 1** and **Figures 1A–C**). Additionally, 11 potential peptides were identified in the domains of NTD (2), C1/2-terminal (4), S1/S2 border (3), HR2 (1) and HR2-TM (1), respectively. We also found five potential peptides in the NSP domain, including ORF1ab (2), ORF10 (1) and ORF7a (2). The same epitope in the NDT domain (^293^LDPLSET^299^), as we found in the NTD domain in our study (^291^CALDPLSETKCTLKS^305^), was reported recently (18). The data together indicate that the S-ECD is a homo-trimeric complex containing multi-epitopes, increasing the neutralizing capacity through various molecular neutralization mechanisms (19).

It is important to find highly conserved peptides/epitopes in the spike protein showing no or very low mutation rates when trying to develop efficient neutralization antibodies and vaccines. Of our novel N-IgY-pAb targeting 20 potent peptide probes, 17 peptides showed no mutations of amino acid residue

(85%) (see **Table 3**). Two were in the NTD domain [^141^LGVYYHKNNKSWMES^155^ (G142D) and

**Table 3:** A summary of residue mutations rates (MRs) of SARS-CoV-2 during the SARS-CoV-2 pandemic according to the report by Vilar and Isom (2021) (1), and https://en.wikipedia.org/wiki/SARS-CoV-2_Omicron_variant.

Type	Higher rate score	Lower rate score		
	MRs > 0.20	MRs = 0.05–0.10	MRs = 0.025–0.05	MRs = 0.01–0.025
Spike	D614G, A222V, L18F, *K417N T478K, L452R P681R, E484Q *G142D	N501, A750, T716, S982, D1118	A262, S477, A570, T716, P681, S982,	S98, P215, *P272, Y453, E583
Nucleocapsid	A220, R203, G204	D3, S235, S194, M234, S194	A376, M234	P67, H145
NSP 12	P323		A185, V776, V720	E254, A656, T739,
NSP 15			T34	K13, R207, T115
NSP 16- 19				R216
NSP 5				K90, L89, G15, G71, P132
NSP9	G50N, L67F		M101	H295
PL protease		A145	A890 (NSP3), P223, P968 (NSP3) MRs: 0.07-0.02	

*Grey box: Three mulations were found in our study.

^261^GAAAYYVGYLQPRTF^275^ (P272)] with very mutation rates (MR.< 0.01-0,025) and one was in the

RBD domain [^411^APGQTGKIADYNYKL^425^ (K417N)] (4,5, https://en.wikipedia.org/wiki/SARSCoV-2_Omicron_variant).

The Spike (S) is a homo-trimeric transmembrane glycoprotein that mediates the viral entry into the host cells (19) as the main target for the development of most of the vaccines (6, 8, 9) or NAbs against SARS-CoV-2. Most of the SARS-CoV-2 neutralizing antibodies in use today are monoclonal antibodies (NmAbs), targeting the spike (S) protein receptor binding domain (RBD), which engages the receptor angiotensin-converting enzyme-2 (ACE2) for viral entry (10, 11). Unfortunately, high mutation rates (MR) were found in the S-protein domain of the SARS-CoV- 2 virus (1) and the mutation residue variability is thus affect protective efficacy. Of the 56 residue mutations found, only three were connected to our novel N-IgY-pAb (3/56, 5.3%) in our study.

Although neutralizing antibodies usually target the RBD, structural model analysis of the trimeric spike (S) protein revealed that the Fab fragment of a humanized monoclonal antibody H014 can recognize a conformational epitope on one side of the open RBD, which only involves protein–protein contacts. The H014 antibody can block SARS-CoV-2 attaching to its host cell receptor. The combination of mAbs can recognize more conservative epitopes and enhance the neutralization activity. Epitope analysis of available NmAbs against SARS-CoV-2 revealed a wide range of cross-protected epitopes (28). Further investigation proved that at least two RBD conformations are involved in the attaching process, being in an “up” and slightly rotated configuration or in a “down” configuration (29). The multiple different antigenic sites, including several RBD epitopes and non-RBD epitopes (30, 31), as well as the S1/S2 cleavage site (polybasic), are essential for S-protein-mediated cell-cell fusion and entry into human lung cells (31, 32) There are seven NmAbs targeting potent epitopes in the NTD domain of the SARS-CoV-2 (29). Of 278 mAbs isolated from three COVID-19-recovered patients, 43.8% were related to the RBD domain and 36.6% to the NTD domain (10). It has been suggested that RBD and NTD together are promising peptides for therapeutic N-mAbs against the COVID-19 virus (30). Strong signals in the NTD domain and at the S1/S2 border were found by proteome microarray (18). The D614G mutation found in the early stages of the pandemic confers greater infectivity and is now the dominant form globally. A higher percentage of the 1-RBD “up” conformation was found in the G614 of the S-protein. The change in conformational kinetics leads to an increase in the efficiency of neutralization of the G614 variant (33, 34).

The downstate surface of residue mutations of S-ECD protein is more conserved for activation of SARS-CoV-2 virus – enhancement versus neutralization by SARS-CoV-2 antibodies (34). Mutations in the RBD and S2 domains (S383C/D985C), which lead to thermodynamic prevalence of closed-down conformation, is a quadruple mutant (A570L/T572I/F855Y/N856I), leading to modifications at the interprotomer contacts between SD1 and S2. It can shift the equilibrium away from the closed-down state, yielding a significant fraction of spike conformations in the open receptor-accessible form (28). In a recent study, mutations were found at S98, D215 and P272 within the N-terminal domain, at N439, Y453 and N501 located in the RBD domain, at A570, S982, T716 and D1118 in the S2 subunit (35), essential regions in the binding to the host cell receptor ACE. The risk of mutations in these positions may reduce the use of Abs/vaccine development. It is suggested that although there are now a series of effective vaccines, the combination of multiple neutralizing antibodies may also be crucial for the treatment and prevention of COVID-19 diseases. However, the new N-IgY-pAb developed in this study only binds to less than 2% of the potent peptides with the risk of mutations (**Table 3**), making the novel N-IgY-pAb extremely useful candidates as a neutralization agent.

The neutralizing and immunodominant sites on the SARS-CoV-2 spike protein is of multiple distinct antigenic sites, as discussed in reviews (31). Using structural analysis, molecular and cellular technologies revealed the special binding affinity between SARS-CoV-2 RBD and its receptor ACE2 as well as the diversity of receptor usage, which explained the complex mechanism behind the high infectivity of SARS-CoV-2 virus at the S-protein S1/S2 boundary (36). With different forms of SARSCoV-2 vaccine being developed to reduce this deadly pandemic, fully human neutralizing antibodies (hNAbs) and humanized neutralizing antibodies had been developed as well. A summarized 500 anti-SARS-CoV-2 neutralizing antibodies and found five potential neutralization sites on the SARS-CoV2 S-protein (37). Thus, polyclonal neutralizing antibodies or multi-specific neutralizing antibodies should be developed to neutralize of SARS-CoV-2 more effectively. It suggested that NAbs integrated into “cocktails” are now in clinical implementation (38). However, such NAbs are rather expensive to produce and the implemented design, production and purification strategies need to be improved.

The passive immunization of the chicken immune system has been studied for more than 100 years (39) and contributed substantially to our understanding of the fundamental concepts of immunology and the development of different immunoglobulin classes (14, 15). The chicken immunoglobulin Y (IgY) is a highly conserved homolog of human immunoglobulin G (IgG) (5, 40), however, IgYs do not bind to human Fc receptors or fix mammalian complement components, and do not trigger potentially dangerous immune responses (14, 15, 37, 41, 42). The high neutralizing IgYs (“cocktail”) are stored in eggs of avian in order to protect their own infants against the endogenous toxic antigens from bacterial/virus (15). Thus, highly neutralizing IgYAbs by the passive immunization system of hens were obtained as compared to the mammal IgGs. Today, some highly neutralizing IgY against SARS-CoV2 for protective and therapeutic agents have been reported (41, 42).

In this study, purified N-IgY-Ab targeting seventeen potent peptides (85%) with highly conserved amino acid residue among 20 peptides (Z-score > 3.0). Despite of three peptides (15%) among the 20 peptides that contain mutation of amino acid residue (Z-score > 3.0), the neutralization effect % of the purified N-IgY-Ab and its “intranasal spray” reach to 98% or more (**Figures 3** and **4**). Our finding of the N-IgY-Ab with high neutralization is further validated by previous reports of the IgY’s benefit. It has further been verified that the chicken immunoglobulin Y (IgY) is a highly conserved homolog of immunoglobulin G (IgG) (40), thus having a high neutralization effect on antigens compared to mammalian IgG (15).

In this study, the novel “intranasal spray” showed favorable stability when stored in a refrigerator (≈4 °C) for at least one year, keeping the high neutralizing effects (**Figures 3** and **4**). Overall, available data up to date confirmed that the IgY antibodies of egg-laying hens are safe, fast-acting, easy to produce, and low cost which can readily be generated in large quantities with minimal environmental harm or infrastructure investment. Thus, the non-parenteral administration, does not have unwanted off-target proinflammatory effects and is nontoxic to humans, it is permitting potential clinical applications in diverse populations and diseases (41, 42), as well as the passive immunotherapy by oral administration of IgY has been used for prophylaxis and treatment of rotavirus diarrhea in human (43). We have prepared a few preventive agents against the SARS-Cov-2 virus. One agent was prepared as a spray for nasal/lung inhalation, and another was designed for external use–for example, washing hands. Now we are planning to use the intranasal spray for clinical studies as protective and therapeutic agent.

Our results confirmed that the N-IgY-pAb have a specific immune response to multiple epitopes within the SARS-CoV-2. It would provide effective material for further developing therapeutic agents or preventive agents against SARS-Cov-2 in the future. A key role for antibody-based of the selected conservative epitope of the peptides or proteins of the SARS-Cov-2 virus for the prevention and treatment of the SARS-Cov-2 virus must be considered. According to our novel N-IgY-pAb targeting 17 potent peptides with conservative residues, we have implemented a therapeutic humanized N-IgY-Abs project for a cocktail of N-IgY-pAb, based on a phage display platform derived from hen eggs. It will be useful for the treatment and prevention of SARS-CoV-2 infection and is a simple, fast and safe approach for treating patients and testing the risk of infected patients effectively. We conclude that our novel NIgY-pAb multi-peptide antibodies neutralized the SARS-CoV-2 effectively. The new findings of 17 conserved potential epitopes are extremely important for the efficient treatment of the SARS-COVID-19 diseases, using a cocktail of antibodies including our N-IgY-pAb.

## Data availability statement

The raw data supporting the conclusions of this article will be made available by the authors, without undue reservation.

## Ethics statement

The animal study was reviewed and approved by Ethics Committee of Xiang Tan Central Hospital of China according to the Experimentation Guidelines (Approval No. 2020-03-001).

## Author contributions

Conception and design of the experiments: JL, TL, AH, XW, JZ, EH, and SS. Data analysis of proteome microarray, interpretation and figures preparation: TL, XW, EH, SS, AH, JL, HL, XY and RZ. Neutralization assay and data analysis: HL, JL, EH, and SS. Preparation, purification and data analysis of the N-IgY Abs: PG, CF and ML. Preparation and writing the final version of the manuscript: SS, EH, AH, JZ. Supervised all process: JL, TL, XW, JZ and EH. All authors contributed to the article and approved the submitted version.
